# Mercator: A modular swarm-dedicated robot platform

**DOI:** 10.1016/j.ohx.2026.e00751

**Published:** 2026-02-20

**Authors:** Miquel Kegeleirs, David Garzón Ramos, Jeanne Szpirer, Cristobal Lara Vera, Guillermo Legarda Herranz, Ilyes Gharbi, Gianpiero Francesca, Mauro Birattari

**Affiliations:** a IRIDIA, Université libre de Bruxelles, Belgium; b LAMS, School of Mechanical and Materials Engineering, University College Dublin, Ireland; c Facultad de Ingeniería en Mecánica y Ciencias de la Producción, Escuela Superior Politécnica del Litoral, ESPOL, Ecuador; d Toyota Motor Europe, Belgium

**Keywords:** Mobile robot, Swarm robotics, ROS, Open-source robot, Differential-drive robot, Autonomous robot

## Abstract

Despite decades of research, swarm robotics still lacks realistic applications. A primary obstacle is the absence of modern, reliable, and affordable robot platforms for experimentation. Existing commercial robots are often outdated or too limited in sensing, computation, and communication. We introduce Mercator, a modular mobile robot purpose-built for contemporary swarm studies in both laboratory and indoor environments. Mercator integrates on-board object and people recognition, short- and long-range obstacle detection, ceiling-based tracking, and local, decentralized communication. By combining these capabilities in a low-cost and extensible package, Mercator enables swarm robotics research to align with modern mobile robotics standards, supporting navigation, mapping, and advanced perception in laboratory settings. This design promotes closer alignment between laboratory swarm experiments and contemporary robotic systems used in real-world settings. The platform has already been used in peer-reviewed scientific work and several master’s theses, and we plan to expand its use in future research and teaching.

## Graphical abstract

**Figure:**
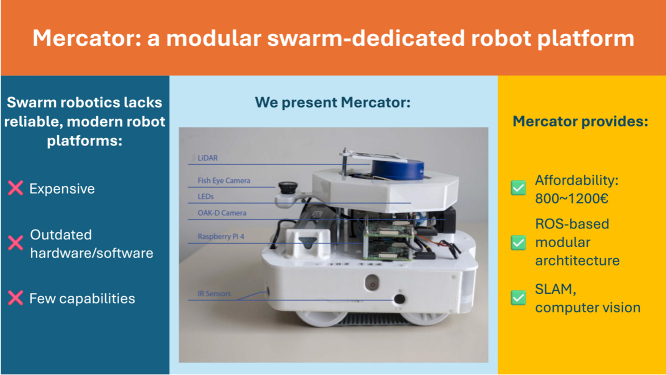

## Specifications table

**Table:** 

**Hardware name**	Mercator
**Subject area**	Engineering and material science
**Hardware type**	Electrical engineering and computer science
**Closest commercial analog**	TurtleBot 4
**Open source license**	Creative Commons Attribution 4.0 International
**Cost of hardware**	€1200
**Source file repository**	https://doi.org/10.5281/zenodo.17399308

## 1 Hardware in context

A major barrier to real world experimentation with robot swarms is the lack of modern, affordable, and reliable experimental robot platforms [1]. Experiments with physical robots remain expensive and time-consuming—challenges that scale with swarm size. Researchers also face logistical constraints: large experimental spaces are often unavailable or costly, and acquiring dozens or hundreds of robots to setup a robot swarm is financially prohibitive. Widely used platforms in single- or multi-robot research — such as AgileX Limo [2], Clearpath Husky [3], or TurtleBot 4 [4] — are typically too large and/or expensive to scale effectively for swarm robotics experiments. This has led to the development of swarm-specific platforms that prioritize low production cost and compactness, often at the expense of sensing, actuation, and processing capabilities.

Among these, the e-puck [5] and the Kilobot [6] are the most prominent, but both remain limited with respect to modern robotics research—manufactured with noisy sensors, unreliable actuators, and outdated hardware. As a result, researchers often rely on abstracted environments and simplified tasks [7] to work around hardware constraints. This sets a discrepancy between swarm robotics’ target use cases — e.g., search-and-rescue, space exploration, and environmental monitoring — and simplified experiments that still dominate much of the literature [1], [7]. Focusing research on limited robots such as the e-puck and the Kilobot prevents adopting the very capabilities that make modern field robotics possible—such as SLAM for enhanced navigation, vision-based perception for scene understanding, and people detection or re-identification for human-centric tasks [8].

In contemporary mobile robotics research, such capabilities are typically given for granted: robotic systems are expected to integrate onboard perception, state estimation, and navigation within standardized and modular software architectures [9]. In practice, these capabilities are also often tightly coupled within a software architecture supported by the Robot Operating System (ROS), which has become the de facto middleware for mobile robotics research and deployment. Compliance with ROS-based workflows enables reproducibility, modular development, and direct reuse of widely adopted software components, and is now a defining characteristic of modern mobile robotic platforms.

A few researchers have devoted effort to modernize legacy swarm robotics platforms. For example, the Pi-puck extension [10] boosts the e-puck’s processing power with a Raspberry Pi; the Xpuck achieves a similar upgrade using a Hardkernel Odroid XU4 [11]; and the ROS-compatible operating system *DeimOS* [12] provides modern software support without any hardware modifications. A similar upgrade is available for the Kilobot [13]. However, these solutions are still bound by the limitations of the original robot design.

The cost/capability ratio is equally critical. The Kilobot is inexpensive, but its sensing, actuation, and processing are extremely limited. More problematic is the e-puck: even though its hardware is outdated, its base price is rather high, and it climbs further when commonly required add-ons — such as the Omnivision Module extension — are included. See Table 1 for costs.

To overcome the combined hurdles of cost, scalability, and outdated sensing/compute in existing swarm platforms, we introduce Mercator—a low-cost, ROS-driven robotic platform specifically designed for advanced swarm robotics experiments. Mercator combines compactness, extensibility, and affordability with modern sensing and computation capabilities. Its main features include:

• A varied suite of sensors and processing modules for SLAM, computer vision, and environment interaction.
• Full compatibility with both ROS1 and ROS2.
• A compact, robust form factor suitable for indoor and urban environments.
• Support for modern mobile robotics research, such as people detection, re-identification, and collaborative navigation.

**Fig. 1:**
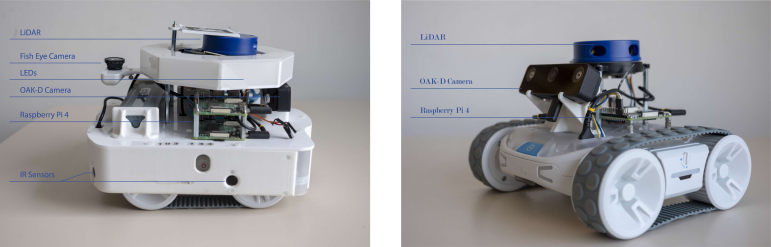
Mercator (left) and Mercator-Lite (right) with their main components.

The platform is available in two configurations (see Fig. 1):

• **Mercator (Standard)**: A fully-equipped version with comprehensive sensing and onboard computing, ideal for vision-based tasks and SLAM.
• **Mercator-Lite (Lite)**: A reduced-cost version with essential sensors, optimized for large-scale swarm deployment and basic swarm behavior testing.

Table 1 compares the two Mercator variants with several commercially available swarm robotics platforms commonly used in research and education. To ensure a fair and focused comparison, we limit our analysis to commercially available platforms widely adopted in swarm research. The comparison focuses on key features relevant to open-source development, modularity, and accessibility. Table 1 details ROS compatibility, ease of customization, availability of design files and code, and relative cost, as follows:

**Table 1:** Comparison of Mercator platforms with common swarm robotics platforms.

Platform	ROS	Customizable	Open Source	Cost (EUR)	Max Speed	Run Time	Size (L×W×H)
**Mercator**	Yes (ROS1/2)	Yes	Yes	∼1200	1 m/s	∼2 h	250 × 260 × 200 mm
**Mercator-Lite**	Yes (ROS1/2)	Yes	Yes	∼850	1 m/s	∼4 h	185 × 216 × 70 mm
e-puck [5]	Via Pi-Puck	Limited	Partial	∼1500+*	0.15 m/s	∼1 h	70 × 70 × 50 mm
Kilobot [6]	No	No	Partial	∼100	0.01 m/s	∼3 h	33 × 33 × 30 mm
TurtleBot3 Burger	Yes (ROS1/2)	Yes	Yes	∼800	0.22 m/s	∼2 h 30	138 × 178 × 191 mm
TurtleBot3 Waffle Pi	Yes (ROS1/2)	Yes	Yes	∼2000+	0.26 m/s	∼2 h	281 × 306 × 141 mm
TurtleBot4	Yes (ROS2)	Yes	Partial	∼3000	0.31 m/s	∼3 h	341 × 339 × 351 mm
Khepera IV [14]	Partial	Limited	No	∼3000+	0.9 m/s	∼2 h	135 × 135 × 70 mm
Thymio II [15]	No	Limited	Yes	∼200	0.14 m/s	∼3 h	110 × 112 × 53 mm

${}^{*}$The indicated price comprises the base e-puck robot and the Pi-puck extension.

• **ROS**: Indicates whether the platform is natively compatible with the Robot Operating System.
• **Customizable**: Denotes the ease with which the robot can be reconfigured or extended.
• **Open source**: Refers to the availability and maintenance of both hardware (e.g., schematics, 3D models) and software (e.g., ROS packages), enabling replication without direct contact with the developers.
• **Cost**: Provides a rough estimate of affordability, crucial for scaling to large swarm sizes.

As shown in Table 1, most swarm robotics platforms are not (fully) open-source and lack native ROS compatibility. They also tend to fall into two categories: (i) expensive robots with limited capabilities due to outdated hardware (e.g., e-puck and Khepera IV), or (ii) affordable robots that are severely limited in functionality (e.g., Kilobot and Thymio II). The TurtleBot family represents the closest commercially available alternative to Mercator. However, it is primarily designed as a general-purpose robotics platform solely focused on mobile robotics. As such, TurtleBots lack native support for robot-to-robot communication and perception, which are fundamental requirements for swarm robotics research. In contrast, Mercator is explicitly designed to support swarm interactions, natively integrating interaction mechanisms well established in the swarm robotics literature — such as color-based signaling for local communication and perception [16] — without requiring additional hardware or substantial platform modification. Custom-built robots — such as Swarmanoid Project’s Footbot [17], Colias [18], and Mona [19] — while valuable for specific tasks, are often designed for narrow applications, lack standardization, and are difficult to reproduce [20]. Even when schematics and assembly instructions are published, duplicating a custom design at scale is often time-intensive and requires advanced technical skills. In contrast, Mercator was designed to bridge this gap by providing a platform that combines the affordability and scalability of simple robots with the sensing, computation, and ROS compatibility of more advanced systems—while remaining easy to assemble using off-the-shelf components and minimal technical expertise, unlike most custom-built alternatives. Its design emphasizes accessibility, low cost, and functional density, enabling researchers to build scalable swarms with advanced capabilities using readily available hardware and open-source software.

## 2 Hardware description

The Mercator platform extends the capabilities of the Sphero RVR+ robot,^2^ a commercially available robot originally designed for educational purposes. Mercator retains the RVR+’s native features — such as differential tracked drive, odometry, an IMU, and a ground color sensor — and further integrates additional computation and sensing modules to enable advanced swarm robotics experiments.

### 2.1 Mercator (standard)

The standard Mercator configuration is engineered for computationally intensive tasks and enhanced perception. It features:

• Processing: Dual Raspberry Pi 4 Model B units.
• Architecture: The primary Raspberry Pi, powered by the RVR+’s internal battery, handles low-level control and runs a ROS driver (i.e., a ROS software stack to communicate with the RVR+ low-level microcontroller). The primary Raspberry Pi interfaces with a rotating LiDAR and eight IR proximity sensors. The secondary Raspberry Pi, powered by an additional RVR battery, is dedicated to vision processing using an OAK-D spatial camera and an upward-facing fisheye camera. This secondary Raspberry Pi also manages a set of RGB LED indicators.
• Sensing and Actuation:
  – 2D rotating LiDAR (YDLIDAR X4) with a 360-degree field of view, used for real-time obstacle detection and mapping.
  – OAK-D W stereo vision camera with onboard AI inference. It provides depth estimation and object recognition capabilities and serves as the robot’s primary visual perception module.
  – Upward-facing fisheye camera (standard Raspberry Pi camera fitted with a wide-angle fisheye lens) for ceiling-based localization.
  – 8 × IR proximity sensors (TeraRanger Multiflex) distributed around the robot perimeter. They provide short-range obstacle detection and proximity awareness.
  – 8 × individually addressable APA RGB LEDs used for robot identification, signaling, and debugging feedback during swarm tasks.
  All modules connect to the Raspberry Pis via USB or GPIO interfaces.
• Software: Ubuntu 20.04 with support for both ROS1 and ROS2. This architecture enables rapid development, modular expansion, and integration with ROS-compatible components.

All components are mounted using lightweight, 3D-printed brackets and laser-cut acrylic. The entire system is designed for reproducibility, using only off-the-shelf parts. The assembly of Mercators is straightforward and typically takes under 3 h, with a total estimated cost of approximately €1200 per robot. Support for the UWB-based communication system is under development, with a dedicated space for the devices already integrated into the design.

### 2.2 Mercator-lite

The **Mercator-Lite** is a lightweight, cost-effective variant optimized for large-scale swarm deployments. It includes:

• A single Raspberry Pi 4.
• A rotating LiDAR (YDLIDAR X4) and an OAK-D W camera.

Despite being endowed with fewer hardware components, Mercator-Lite maintains full software compatibility with the standard Mercator. This enables Mercator and Mercator-Lite to be operated in mixed heterogeneous robot swarms. Unlike Mercator, Mercator-Lite can be built on earlier revisions of the Sphero RVR+ (e.g., RVR), as a means to reduce costing.^3^ Mercator-Lite can be assembled in under 1 h, with an estimated cost of approximately €850 per robot.

### 2.3 Software stack and modularity

Mercator software architecture is modular, ROS-native, and designed for extensibility. Both Raspberry Pis run Ubuntu 20.04 and support a wide array of robotics libraries. Each sensor and actuator is managed by dedicated ROS nodes, allowing easy customization and integration of additional hardware. In addition, ROS facilitates fusing readings from the LiDAR and depth camera, which is used for detecting other robots and estimating their relative pose (similarly to the range-and-bearing module of the e-puck).

The system adheres to standard ROS conventions, using publish–subscribe topics with standard message types. This approach facilitates debugging, modular reuse, and data logging. To address inconsistencies in the RVR+ color sensor — caused by overly fine-grained raw RGB outputs — a dedicated ROS node simplifies and stabilizes readings by mapping them to predefined color labels using a nearest-neighbor classifier.

Mercator supports both ROS1 (Noetic) and ROS2 (Foxy). ROS1 is used as the primary development environment due to its long-standing stability, extensive ecosystem, and mature support for widely adopted packages for SLAM, navigation, and sensor interfacing, which remain central to many experimental robotics workflows. Although ROS1 has formally reached end-of-life and Ubuntu 20.04 is approaching end-of-life, both continue to be widely used in research laboratories. Their adoption therefore represents a pragmatic choice that prioritizes experimental robustness, reproducibility, and compatibility with existing hardware and software stacks over early migration to newer but still evolving frameworks. Importantly, the system architecture is designed to remain forward-compatible with ROS2. Core components are modular and abstracted from middleware-specific implementations, enabling a gradual transition toward ROS2-based communication and more scalable multi-robot frameworks as these mature and become standard practice. Interoperability between ROS1 and ROS2 is also supported through established bridging mechanisms (e.g., the ros1_bridge package^4^), allowing mixed deployments and incremental migration.

Finally, it is important to note that the swarm experiments examples presented in this paper rely primarily on local, on-board perception and decentralized coordination—as this is a common setup in swarm robotics literature. The collective behavior of the swarm emerges solely from the local interaction that robots have with their peers and their environment. That is, the robots operate autonomously without centralized state estimation or structured inter-robot data exchange, and therefore do not directly benefit from the more advanced multi-robot system features introduced in ROS2 (i.e., multi-robot communication over topics). In this context, deploying a ROS1 architecture in each robot provides all required functionality without imposing additional architectural complexity. Yet, we are currently developing a ROS2 equivalent of the software that is available for Mercator in ROS1. Future work will leverage ROS2 middleware to support the exchange of more structured information between robots, enabling higher-level coordination mechanisms such as identity persistence and richer collective perception [21], [22].

### 2.4 Simulation compatibility

Mercator is fully integrated into the ARGoS3 simulation environment [23], featuring accurate modeling of actuators and sensors via the argos-rvr plugin. Sensor noise profiles are based on real-world measurements, supporting high-fidelity simulation-to-hardware transfer.

The platform is also compatible with AutoMoDe-RVR, a control software design framework tailored for Mercator that extends the family of methods AutoMoDe [24], [25]. AutoMoDe-RVR allows the optimization-based design of control software for Mercator via the design and simulation of probabilistic finite state machine (PFSM) controllers. Although ARGoS handles control natively without ROS, software developed in simulation can be transferred to physical Mercator units with minimal modification. Control software produced with AutoMoDe has been successfully ported from the e-puck to Mercator hardware with minimal adjustments [26]—showing transferability of standard swarm behaviors across platforms. We also provide support for frameworks to design control software for Mercator via neuroevolutionary robotics (NEAT [27], CMA-ES [28], and xNES [29]). See Table 3 (S1–S10) for details on the software components.

### 2.5 Key features

• **Modular and ROS-native:** Customizable using sensor-specific ROS nodes.
• **Seamless simulation-to-hardware pipeline:** Compatible with ARGoS3 and AutoMoDe.
• **Cost-effective:** Comparable in cost to legacy platforms like the e-puck, with significantly enhanced capabilities.
• **Advanced functionality:** SLAM, computer vision, ceiling localization, and people tracking.
• **Heterogeneous swarm support:** Unified operation across standard and Lite models.
• **Accessible design:** Assembled from off-the-shelf parts with 3D-printed and laser-cut mounts.
• **Detailed specifications:** See Table 2 for a complete comparison of the two Mercator variants.

In summary, Mercator can support robotics research by:

**Table 2:** Key specifications of Mercator and Mercator-Lite.

Feature	Mercator	Mercator-Lite
Base platform	Sphero RVR+	Sphero RVR or RVR+
Processing units	2×Raspberry Pi 4B	1×Raspberry Pi 4B
Devices	LiDAR, OAK-D W, fisheye, IR, LEDs	LiDAR, OAK-D W
Software	Ubuntu 20.04 + ROS1/2	Ubuntu 20.04 + ROS1/2
Power supply	Internal + external battery	Internal battery
Estimated cost	∼€1200	∼€850
Size (L × W × H)	250 × 260 × 200 mm	185 × 216 × 70 mm
Weight	2.8 kg	1.5 kg
Open source license	CC BY 4.0	CC BY 4.0
Repository	Zenodo	Zenodo

• Offering an affordable and reproducible platform for repeatable swarm experiments with modern sensing and standardized ROS-based software.
• Enabling real-world validation of collaborative SLAM, swarm perception, and decentralized coordination algorithms.
• Serving as a hands-on system for education and training in contemporary mobile robotics workflows.
• Facilitating studies of human–robot interaction using onboard vision-based perception.
• Bridging swarm robotics research with modern mobile robotics standards through transferability of algorithms across platforms.

### 2.6 Architecture of a swarm robotics experiment with a group of Mercators

**Fig. 2:**
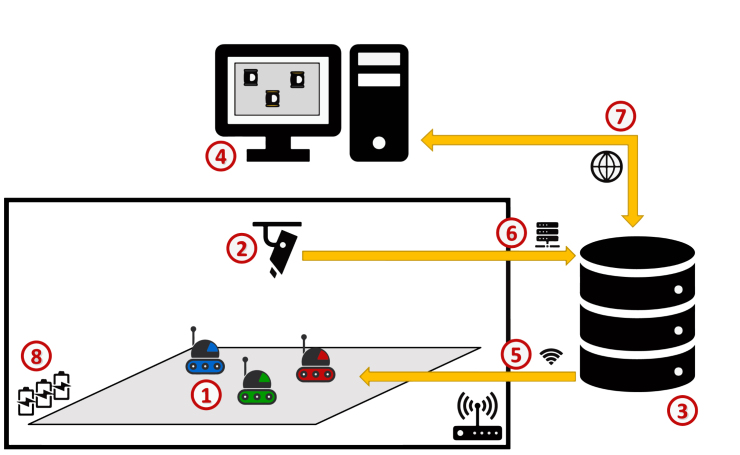
Typical architecture of a swarm robotics experiment with Mercators. The figure illustrates the (1) Mercators, (2) tracking system, (3) experiments server, (4) workstation, (5) wireless router, (6) experiment information streamed to the server, (7) access to experiments server, and (8) charging docks for Mercator.

A typical swarm experiment involving Mercators is structured as follows (see Fig. 2). A group of Mercators is deployed in an experimental environment (1), which may include obstacles, mission-related objects, or other robots. A wireless router provides network connectivity (5), allowing to deploy control software into the robots and receive experiment instructions — typically to initialize or start a run — from a experiments server (3), which acts as an operation and monitoring interface for the experimental infrastructure. The environment is monitored by an external tracking system (1) — for example, Tycho [30] — which estimates the state of each robot throughout the experiment. This information is streamed (6) to the server for logging, visualization, and post-experimental analysis. The tracking system does not communicate directly with the robots and does not influence their behavior during execution. Researchers interact with the system through a workstation (4), which provides access to the server, the tracking data, and experiment control tools either locally or for from the web (7). Finally, sufficient batteries and/or charging docks should be available (8) to enable rapid turnaround between experimental runs.

Typically, the experimental workflow proceeds as follows. Required materials, such as robot control software and configuration files, are deployed to Mercators via secure shell (SSH). On each robot, a ROS master node is launched together with the RVR driver and the required sensing and actuation components (e.g., LiDAR, cameras, LEDs). Prior to each run, robots are positioned in the environment either manually or automatically using the tracking system—i.e., driving the robots to desired starting positions. The experiment is then initiated by starting the robots’ control software. During execution, Mercators operate fully autonomously, relying exclusively on onboard sensing, local computation, and decentralized interaction mechanisms. As noted in past sections, robot-to-robot coordination is currently achieved through local perception and signaling (e.g., by using the colors of Mercator’s LEDs module), rather than through centralized communication or shared global state. The tracking system provides real-time feedback to the researchers but does not participate in the control loop. It is used to track the motion of the robots and use this information to compute their performance in the task at hand. At the end of a run, robots are halted, batteries are replaced if necessary, and the procedure can be repeated for subsequent trials.

## 3 Design files summary

This section summarizes the mechanical, electronic, and software components required to replicate the Mercator platform. Unless otherwise noted, the descriptions and design files refer to the full Mercator configuration (Standard version). A separate subsection at the end covers the Mercator-Lite variant, which uses a reduced list of components.

### 3.1 Mercator design

The mechanical structure of Mercator is composed of modular elements mounted on the Sphero RVR+. Components are fabricated using a combination of laser-cut acrylic and 3D-printed PLA or PETG parts. The design emphasizes robustness and ease of assembly.

The structure includes (see Table 3 P1–P8 for corresponding files):

• **P1 – Base plate:** A laser-cut platform that attaches securely to the top of the RVR+ and serves as the foundation for all other elements.
• **P2 – Battery rack:** A combination of laser-cut (P2.1) and 3D-printed (P2.2-2.3) elements mounted at the rear of the robot for housing an external RVR battery, used to power the secondary Raspberry Pi and camera modules.
• **P3 – Fuse case:** A 3D-printed case (P3.1) and laser-cut lid (P3.2) that protect the voltage DC-DC regulator and the fuse connected to the external battery.
• **P4 – Sensor tower:** An elevated frame composed of laser-cut (P4.1-4.4) and 3D-printed parts (P4.5-4.6), mounted on four metal spacers, and designed to carry the rotating LiDAR and a RGB LED ring. A bracket positioned at the rear of the tower holds the upward-facing fisheye camera. In addition, a flat support for a QR tag identifier is positioned above the LIDAR with the help of spacers.
• **P5 – Camera mount:** A front-facing, 3D-printed bracket that positions the OAK-D W camera at a fixed height and orientation to ensure consistent stereo vision and depth perception.
• **P6 – Camera mount (Lite):** A front-facing, 3D-printed bracket for the Mercator-Lite that positions the OAK-D W camera at a fixed height and orientation to ensure consistent stereo vision and depth perception.
• **P7 – Sensor belt/bumper:** A 3D-printed ring (P7.1-7.3) encircling the robot to mount eight IR proximity sensors. The front-left and front-right corners (P7.4) are laser cut in flexible material. It also acts as a protective bumper during collisions. A side segment of the belt, which covers access to the RVR+’s internal battery compartment, is magnetically attached to the platform to allow quick removal and battery replacement without tools.
• **P8 – UWB module bay:** A 3D-printed case and laser-cut lid at the rear of the robot for optional integration of a UWB communication device.

The rest of Table 3 lists the main electronics (P9–P15) and software (S1–S10) components.

**Table 3:** Mechanical design files (P1–P8), and main electronic (P9–P15) and software (S1–S10) components.

Designator	Description	Type	Open s. license	Files location	Notes
P1	Base plate	DXF	CC BY 4.0	Zenodo	
P2	Battery rack	STL + DXF	CC BY 4.0	Zenodo	
P2.1	Base rail	DXF	CC BY 4.0		
P2.2	Support (button side)	STL	CC BY 4.0		
P2.3	Support (connector side)	STL	CC BY 4.0		
P3	Fuse case	STL + DXF	CC BY 4.0	Zenodo	
P3.1	Case	STL	CC BY 4.0		
P3.2	Lid	DXF	CC BY 4.0		
P4	Sensor tower	STL + DXF	CC BY 4.0	Zenodo	
P4.1	Base plate	DXF	CC BY 4.0		
P4.2	Cover	DXF	CC BY 4.0		
P4.3	Side windows (8)	DXF	CC BY 4.0		
P4.4	Tag support	DXF	CC BY 4.0		
P4.5	LEDs support	STL	CC BY 4.0		
P4.6	Fisheye camera support	STL	CC BY 4.0		
P5	Camera mount	STL	CC BY 4.0	Zenodo	
P6	Camera mount (Lite)	STL	CC BY 4.0	Zenodo	
P7	Bumper/Sensors belt	STL	CC BY 4.0	Zenodo	
P7.1	Right part	STL	CC BY 4.0		
P7.2	Left part	STL	CC BY 4.0		
P7.3	Front part	STL	CC BY 4.0		
P7.4	Corners (2)	DXF	CC BY 4.0		
P8	UWB module bay	STL + DXF	CC BY 4.0	Zenodo	
P8.1	Case	STL	CC BY 4.0		
P8.2	Lid	DXF	CC BY 4.0		

P9	Raspberry Pi 4 (4GB)	Electronics			M33 in BoM
P10	YDLIDAR X4	Electronics			M35 in BoM
P11	OAK-D W stereo camera	Electronics			M36 in BoM
P12	LattePanda camera + fisheye lens	Electronics			M37 and M38 in BoM
P13	TeraRanger Multiflex IR sensors	Electronics			M39 in BoM
P14	LED Strip APA 102	Electronics			M40 in BoM
P15	RVR official battery	Electronics			M32 in BoM

S1	ARGoS3-RVR	Git repo	MIT	GitHub	Plugin for the ARGoS3 simulator
S2	AutoMoDe-RVR	Git repo	MIT	GitHub	AutoMoDe version for Mercator
S3	Reference model	Git repo	MIT	GitHub	Reference model for AutoMoDe
S4	Loop functions	Git repo	MIT	GitHub	ARGoS3 loop functions for AutoMoDe
S5	NEAT-RVR	Git repo	MIT	GitHub	NEAT version for Mercator
S6	Pagmo-RVR	Git repo	MIT	GitHub	C-MAS and xNES versions for Mercator
S7	Drivers and utility	Git repo	MIT	GitHub	ROS nodes for the main driver and sensors
S8	SD image (RPi primary)	Image	CC BY 4.0	Zenodo	Pre-built image for main control and LIDAR
S9	SD image (RPi secondary)	Image	CC BY 4.0	Zenodo	Pre-built image for vision stack + camera nodes
S10	SD image (Lite)	Image	CC BY 4.0	Zenodo	Pre-built image for Mercator-Lite

### 3.2 Mercator-lite variant

Mercator-Lite reuses the same software and provides a reduced list of hardware components—see Table 4. Components are mounted directly on the RVR/RVR+ and a dedicated 3D-printed bracket (P6) is designed for the OAK-D camera.

**Table 4:** Summary of components and resources for the Mercator-Lite variant (refer to Table 3 for part descriptions).

Designator	Component	Notes
P9	Raspberry Pi 4	Main compute unit
P10	YDLIDAR X4	2D LiDAR for obstacle detection
P11	OAK-D W Camera	Stereo vision and depth inference
P6	Camera mount (Lite)	3D-printed bracket for OAK-D
S10	SD image (Lite)	Pre-built image for Mercator-Lite

## 4 Bill of materials

Table 5 provides a summary of the bill of materials for the standard Mercator version. Table 6 provides a summary of the bill of materials for Mercator-Lite. Both bills of materials report only the cost of hardware components and do not account for labor costs (e.g., purchasing, fabrication, and assembly) or for access to equipment such as 3D printers, laser cutters, or soldering tools. These additional costs depend on local infrastructure and available resources and should therefore be estimated separately by the user.

**Table 5:** Comprehensive bill of materials (Mercator).

Designator	Name	Qty	Unit cost (€)	Total cost (€)	Source of material	Material type
M1	M3 8 mm Nylon Countersunk Bolts	28	0.30	8.40	https://befr.rs-online.com/web/	Polymer
M2	M3 16 mm Nylon Countersunk Bolts	9	0.35	3.15	https://befr.rs-online.com/web/	Polymer
M3	M3 12 mm Nylon Countersunk Bolts	2	0.30	0.60	https://befr.rs-online.com/web/	Polymer
M4	M3 6 mm Stainless Steel Bolts	2	0.09	0.18	https://befr.rs-online.com/web/	Metal
M5	M3 12 mm Stainless Steel Bolts	13	0.11	1.10	https://befr.rs-online.com/web/	Metal
M6	M3 25 mm Stainless Steel Bolts	3	0.15	0.45	https://befr.rs-online.com/web/	Metal
M7	M4 6 mm Stainless Steel Bolts	2	0.13	0.26	https://befr.rs-online.com/web/	Metal
M8	M3 Washers	8	0.08	0.64	https://befr.rs-online.com/web/	Metal
M9	M3 Stainless Steel Nuts	44	0.09	3.96	https://befr.rs-online.com/web/	Metal
M10	M3 Nylon Nuts	7	0.29	2.03	https://befr.rs-online.com/web/	Polymer
M11	M3 Locknuts	25	0.10	2.50	https://befr.rs-online.com/web/	Metal
M12	M3 6 mm Brass Spacers	4	0.48	1.92	https://befr.rs-online.com/web/	Metal
M13	M3 25 mm Stainless Steel Spacers	12	0.43	5.16	https://befr.rs-online.com/web/	Metal
M14	5 mm x 3 mm Neodymium Magnets 0.55 kg	3	1.37	4.11	https://befr.rs-online.com/web/	Composite
M15	5 mm x 5 mm Neodymium Magnets 0.77 kg	3	1.70	5.10	https://befr.rs-online.com/web/	Composite
M16	Step Down Voltage DC-DC Regulator	1	9.90	9.90	https://whadda.com/	Semiconductor
M17	Fusible Support	1	0.45	0.45	https://befr.rs-online.com/web/	Other
M18	3A Fast Fusible	1	0.44	0.44	https://befr.rs-online.com/web/	Other
M19	Switch On/Off	1	3.71	3.71	https://befr.rs-online.com/web/	Other
M20	Springs for Battery Connections	2	0.62	1.24	https://befr.rs-online.com/web/	Metal
M21	Female Connector Housing, 2 mm Pitch, 2 Way, 1 Row	2	0.07	0.14	https://befr.rs-online.com/web/	Polymer
M22	Male Crimp Connector Housing, 2 mm Pitch, 2 Way, 1 Row	2	0.18	0.36	https://befr.rs-online.com/web/	Polymer
M23	micro-USB to USB cable (RPi to LIDAR)	1	1.45	1.45	https://befr.rs-online.com/web/	Other
M24	Right-angle USB-C to USB cable (RPi to RVR)	2	13.67	27.34	https://befr.rs-online.com/web/	Other
M25	Ethernet Cable (Raspberry to Raspberry)	1	1.80	1.80	https://befr.rs-online.com/web/	Other
M26	Jumpers Female-Female Connection (Raspberry to RVR and LEDs)	7	0.22	1.54	https://befr.rs-online.com/web/	Other
M27	PLA 3D printer filament	1^a^	29.36	29.36	https://befr.rs-online.com/web/	Polymer
M28	PLEXIGLAS © LED, White WH14 GT, 3 mm	1^a^	29.70	29.70	https://www.plexiglas-shop.com/	Polymer
M29	PLEXIGLAS © XT, White WN297 GT, 3 mm	1^a^	20.90	20.90	https://www.plexiglas-shop.com/	Polymer
M30	Soft PVC strip 100 × 1.2 mm	1^a^	2.03	2.03	https://www.superplastic.be/	Polymer
M31	Sphero RVR+	1	349	349	https://www.eduwinkel.nl/	Other
M32	RVR Battery	2^b^	70	70	https://www.eduwinkel.nl/	Other
M33	Raspberry Pi 4B 8Go	2	78.71	157.42	https://befr.rs-online.com/web/	Semiconductor
M34	Micro SD card (at least 32GB)	2	5.68	11.32	https://befr.rs-online.com/web/	Other
M35	YDLIDAR X4	1	75.90	75.90	https://6eu.robotshop.com/	Semiconductor
M36	OAK-D W Camera	1	331.55	331.55	https://www.mouser.be/	Semiconductor
M37	Camera UVC 5MP USB LattePanda	1	27.50	27.50	https://6eu.robotshop.com/	Semiconductor
M38	Fisheye lens for mobile phone	1	4.46	4.46	https://www.amazon.com/	Other
M39	TeraRanger Multiflex 8 Sensor Kit	1	46.95	46.95	https://eu.robotshop.com/	Semiconductor
M40	LED Strip APA 102	25	0.29	7.25	https://opencircuit.shop/	Semiconductor

a Represents one unit of the product; however, constructing a single Mercator does not require its full amount.

b One battery is provided with the Sphero RVR+, but Mercator requires an additional battery.

**Table 6:** Comprehensive bill of materials (Mercator-Lite).

Designator	Name	Qty	Unit cost (€)	Total cost (€)	Source of material	Material type
M5	M3 12 mm Stainless Steel Bolts	14	0.13	1.82	https://befr.rs-online.com/web/	Metal
M8	M3 Washers	28	0.08	2.24	https://befr.rs-online.com/web/	Metal
M9	M3 Stainless Steel Nuts	4	0.09	0.36	https://befr.rs-online.com/web/	Metal
M11	M3 Locknuts	18	0.10	1.80	https://befr.rs-online.com/web/	Metal
M12	M3 6 mm Brass Spacers	4	0.48	1.92	https://befr.rs-online.com/web/	Metal
M13	M3 25 mm Stainless Steel Spacers	8	0.43	3.44	https://befr.rs-online.com/web/	Metal
M23	micro-USB to USB cable (RPi to LIDAR)	1	1.45	1.45	https://befr.rs-online.com/web/	Other
M24	Right-angle USB-C to USB cable (RPi to RVR)	1	13.67	13.67	https://befr.rs-online.com/web/	Other
M26	Jumpers Female-Female Connection (Raspberry to RVR)	3	0.22	0.66	https://befr.rs-online.com/web/	Other
M27	PLA 3D printer filament	1^a^	29.36	29.36	https://befr.rs-online.com/web/	Polymer
M31	Sphero RVR+	1	349	349	https://www.eduwinkel.nl/	Other
M33	Raspberry Pi 4B 8Go	1	78.71	78.71	https://befr.rs-online.com/web/	Semiconductor
M34	Micro SD card (at least 32GB)	1	5.68	5.68	https://befr.rs-online.com/web/	Other
M35	YDLIDAR X4	1	75.90	75.90	https://6eu.robotshop.com/	Semiconductor
M36	OAK-D W Camera	1	331.55	331.55	https://www.mouser.be/	Semiconductor

a Represents one unit of the product; however, constructing a single Mercator-Lite does not require its full amount.

## 5 Build instructions

**Fig. 3:**
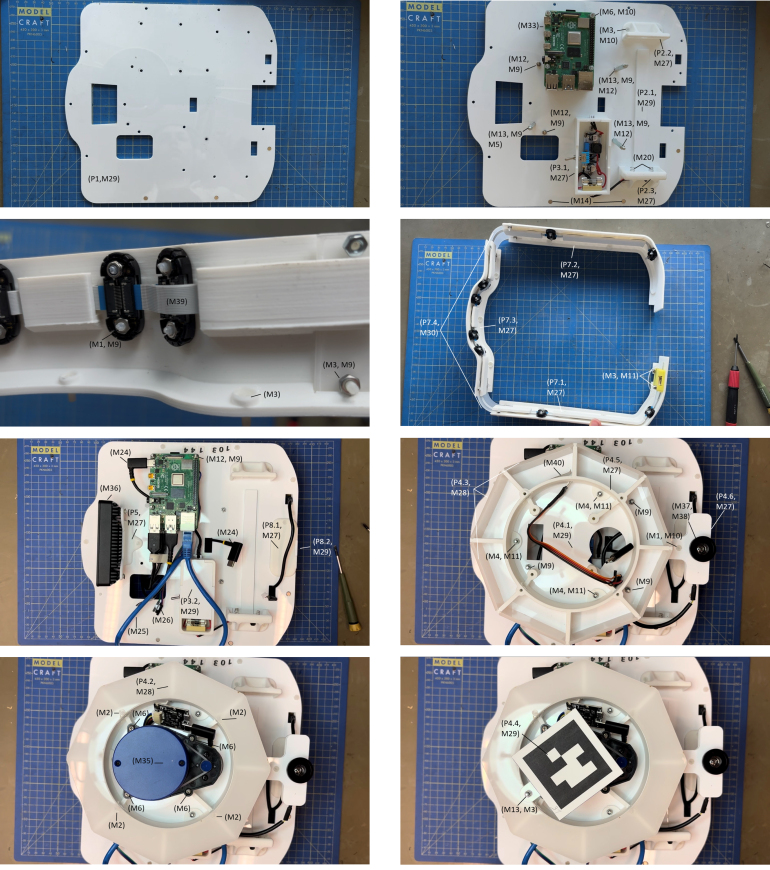
Main assembly steps annotated with materials.

Once the materials described in Table 5 are available, the subsequent task is to produce the mechanical parts (see Table 3 P1–P8) and assemble the robot. The tools required for this process are a screwdriver, pliers, a 3D printer, and a laser cutter. A few steps require some soldering and wire stripping, but no specialized workshop equipment is strictly necessary. The assembly steps described below follow the Mercator construction demo videos,^5^ which serve as a visual guide throughout the process. In addition, Fig. 3 illustrates the main steps, with annotations identifying the relevant components.

### 5.1 Mechanical parts production

The pieces listed in Table 3 (P1–P8) must be produced by 3D printing and laser cutting. 3D-printed pieces include P2.2, P2.3, P3.1, P4.5, p4.6, P5, P7.1, P7.2, P7.3, and P8.1. They can be produced using regular PLA (M27). We selected white PLA so that the surface the bumper (P7) reflects rather than absorbs light, improving its detectability by the IR sensors on other Mercators. Laser-cut pieces include P1, P2.1, P3.2, P4.1, P4.2, P4.3, P4.4, P7.4, and P8.2. The cover and the windows of the sensor tower (P4.2 and P4.3) must be produced with semi-translucent material to ensure proper color diffusion (M28). The corners of the bumper (P7.4) must be produced with flexible material (M30). The other parts can be produced with white Plexiglas © (M29). We selected this material for its resilience and favorable aesthetic properties, but any other rigid material (e.g., MDF) can be used to reduce costs. Similarly, nylon bolts and nuts (M1, M2, M3, and M10) have been selected to match visually with Plexiglas © parts, but can be replaced by regular steel equivalents to reduce costs.

### 5.2 Mercator assembly

This section describes the installation of key structural and functional components on the base plate, including the sensor belt, power supply system, computing units, and mounting elements. These steps are carried out before attaching the base to the Sphero RVR+ and are essential for ensuring proper layout, accessibility, and cable organization. Most elements are first assembled and connected to the base plate, with the exception of the sensor tower (Sections 5.2.1, 5.2.3, and 5.2.2). All necessary cables are then properly routed and connected to the electronic devices (Section 5.2.5). The sensor tower is assembled and connected last to the plate (Section 5.2.4).

#### 5.2.1 General assembly

The following steps describe how to assemble and attach the different elements to the laser-cut base plate (P1).

1. **Battery rack (P2):** Solder a short insulated hook-up wire to each of the two springs (M20). Position the springs in the dedicated space of one of the support (P2.3), pass the wires through the rear hole and fix the springs with glue. Attach a male crimp connector (M22) to the wires. Position the laser-cut rail (P2.1) and the 3D-printed battery supports (P2.2 and P2.3) to the rear section of the base plate and secure them using the matching screw holes (M2, M3, M11). P2.3 is positioned on the top of P2.1 and both are secured together. This will house the external RVR battery (P15).
2. **Fuse case (P3):** Assemble the fuse case (see Section 5.2.2 and position it adjacent to the battery mount. Secure it using the matching screw holes (M4, M11) on the base plate.
3. **Sensor tower (assembly) (P4):** Assemble the sensor tower (see Section 5.2.4 but do not attach to the base plate yet).
4. **Camera mount (P5):** Attach the OAK-D W camera (P11) to the 3D-printed mount (P5) using appropriate bolts (M7). Then, fix the mount to the front of the plate and install the camera in a stable forward-facing position. Secure it using the matching screw hole (M2, M11).
5. **Bumper (P7):** Insert neodymium magnets (M14) in the corresponding holes in the left part of the base plate and glue them. They should align with the magnets on the left part of the bumper. Assemble the bumper (see Section 5.2.3) and attach it with nylon nuts (M10) to the base plate using matching screw holes.
6. **UWB module bay (P8):** If UWB capabilities are required, secure the UWB modules inside the module bay (M6, M11) and attach the bay on the rear side of the base plate (M2, M11).
7. **Raspberry Pi boards (P9):** Mount both Raspberry Pi 4 boards on the right side of the base plate. The bottom one will serve as the primary control unit, the upper one for visual processing. First, secure the bottom one using bolts (M5) pointing upward. It is recommended to place nuts (M9) between the board and the plate to leave some space for pins and soldering protruding out of the bottom of the board. Then, attach spacers (M13) to the bolts and secure the upper Raspberry Pi with locknuts (M11).
8. **Mounting screw preparation:** Insert and loosely secure the bolts and spacers (M5, M12) that will later be used to attach the base plate and the sensor tower to the Sphero RVR+ mounting plate. These also serve as anchor points for organizing and guiding cables underneath the base, contributing to a clean and efficient cable layout.
9. **Connections:** Proceed with the setup of internal connections (see Section 5.2.5). It is easier to connect the sensor tower once it has been secured in the next step. Also, do not connect cables to the RVR+ yet.
10. **Sensor tower (securing):** Secure the sensor tower in the center of the plate with the bolts and spacers prepared at step 3 and connect the dedicated cables to the LEDs and LiDAR.
11. **RVR+:** Remove the mounting plate of the RVR+ and secure it to the base plate using bolts and spacers prepared at step 7, additional ones (M5, M12), and locknuts (M11). It is recommended to place washers (M8) between the RVR+ mounting plate and other elements to protect it from wear and tear. Once everything is tightly secured, connect the bottom Rasperry Pi to the RVR+ following instructions in Section 5.2.5.

#### 5.2.2 Fuse case assembly

The fuse case protects the fuse and voltage regulator monitoring the additional battery used to power the upper Raspberry Pi. Its assembly is the sole part of the building process requiring a bit of soldering, with the exception of the connectors for the battery rack.

1. Prepare 6 short pieces of insulated hook-up wire.
2. Attach a wire to the right pin of a female connector (M21) and to the IN- pin of the voltage regulator (M16). Attach another wire to the left pin of the connector and to one of the pin of the fusible support (M17)
3. Attach a wire to the right pin of the on/off switch (M19) and to the other pin of the fusible support (M17). Attach another wire to the center pin of the switch and to the IN+ pin of the voltage regulator (M16).
4. Attach a wire to the right pin of the second female connector (M21) and to the OUT- pin of the voltage regulator (M16). Attach another wire to the left pin of the connector and to the OUT+ pin of the voltage regulator. The wires should be crossed.
5. Install the components into the 3D-printed fuse case (P3.1) and secure them:
  • Secure the fusible support to the top part of the case using a nylon bolt (M1).
  • Insert the connector wired to the fusible support in the hole right beneath it so that it is accessible from outside the fuse case, and glue it.
  • Similarly, insert the other connector in the opposite hole and glue it.
  • Detach the top nuts of the on/off switch to be able to pass the switch in the hole on the side of the case, then secure it again with the nuts.
  • Place the fusible (M18) in its support.
6. Close the case with its laser-cut lid (P3.2). The lid should hold in place without glue to allow easy access to the fusible for maintenance.
7. Prepare the cable to connect the fuse case to the top Raspberry Pi: cut the USB-A end of a USA-A to USB-C cable (M24) to extract the two wires and attach the wires to a male crimp connector (M22).

#### 5.2.3 Bumper assembly

The bumper is designed both to serve as a protection against collision and to support the proximity IR sensors (p13) of Mercator.

1. Prepare the IR sensors as specified in the sensor user guide: assemble the eight sensors into a continuous strip ending with the main connector.
2. Insert the pairs of bolts (M1) that will hold the eight sensors (16 bolts in total) in the three 3D-printed parts of the bumper (P7.1, P7.2, and P7.3). These serve as guides while positioning the sensors.
3. Loosely assemble the three parts using the two laser-cut corners (P7.4) and bolts (M1). Do not fully tighten yet to leave clearance for installing the sensors.
4. Install the sensors:
  (a) Start at the connector end (right side), and secure it with nuts (M9), orienting the Micro-USB toward the cable pass-through in the bumper.
  (b) Secure the first sensor with dedicated nuts (M9).
  (c) Route the data bus along the inner face of the bumper, seating it in the designated sockets. Use the pre-placed bolts as temporary supports while aligning the wiring and subsequent sensors.
  (d) Secure each sensor with its corresponding pair of nuts (M9).
5. Fully tighten the corners that were left slightly loose with nuts (M9).
6. Install flat nylon bolts (M1) facing up on the upper side of the front and right parts of the bumper so they mate with the base plate; apply a small amount of glue to lock them in place and ease the process of attaching the bumper to the base plate.
7. Place the three neodymium magnets (M15) in their dedicated sockets in the left part of the bumper and secure them with glue.

#### 5.2.4 Sensor tower assembly

The sensor tower is designed to elevate and support key perception modules, including the rotating LiDAR (P10) and the upward-facing fisheye camera (P12). This configuration ensures a clear 360°horizontal scan for the LiDAR and enables the fisheye camera to capture ceiling features, which are essential for indoor localization and mapping tasks. The spacers that support the tag-identifier top plate slightly occlude the LiDAR’s field of view. Because the assembly is mounted at the rear of the robot, the impact on data quality is minimal. Moreover, if no tag-based identification is needed — or if an alternative system is used — the plate and spacers can be removed entirely. It is recommended to attach the tower to the base plate just before attaching the plate to the Sphero RVR+, after most electronic connections and cable routing have been done.

1. **Tower support:** Secure the metal spacers (M11, M13) onto the laser-cut base plate of Mercator (P1) at the three designated mounting points. Attach an additional spacer to each of these spacers. They will serve as the structural support for the sensor tower.
2. **LEDs part 1:** Insert the LED strip (P14) in the dedicated space of the LEDs support (P4.5), around the central circular part. Make sure that the connecting part of the strip aligns with the hole provided to allow the 4 jumpers (M26) to pass through and connect them already. Once correctly positioned, you may glue the strip to fix it permanently.
3. **Tower base plate:** With bolts and locknuts (M5, M11), secure the LEDs support (P4.5) to the tower base plate (P4.1), pass the jumpers of the LEDs through the rectangular hole of the tower base plate, then secure the plate to the three supports installed at step 1.
4. **LEDs part 2:** Insert the 8 side windows (p4.3) in their dedicated space of the LEDs support (P4.5). They should hold in place without glue, but you may add some. Then, fix the cover (P4.2) with bolts (M5).
5. **LiDAR:** Position the LiDAR (P10) with its motor aligned with the dedicated hole of the tower base plate (P4.1) and secure it with bolts (M5). Connect the control chip to the LiDAR and to the cable (M23) used for the connection with the bottom Raspberry Pi. Pass the cable through the rectangular hole of the tower base plate, along with the LEDs’ jumpers. The control chip may stay loose where it is as it should not interfere with other components.
6. **Fisheye camera (P12):** Place the LattePanda camera (M39) in the fisheye camera support (P4.6) with the connector side aligned with the dedicated hole, then fix the support beneath the small protruding part of the tower base plate (P4.1) with bolt and locknut (M2, M11). Insert the fisheye lens (M40) in the dedicated hole of the plate. It should hold in place, but you may glue it to fix it permanently.
7. **ID tag:** Attach two spacers (M13) together and secure them to the tower base plate with the remaining screw hole near the rear of the LiDAR (M5, M11). Print your custom ID tag and glue it to the tag support (P4.4). Position the tag above the LiDAER and secure it to the spacers with a locknut (M11).

#### 5.2.5 Electronics connections

**Fig. 4:**
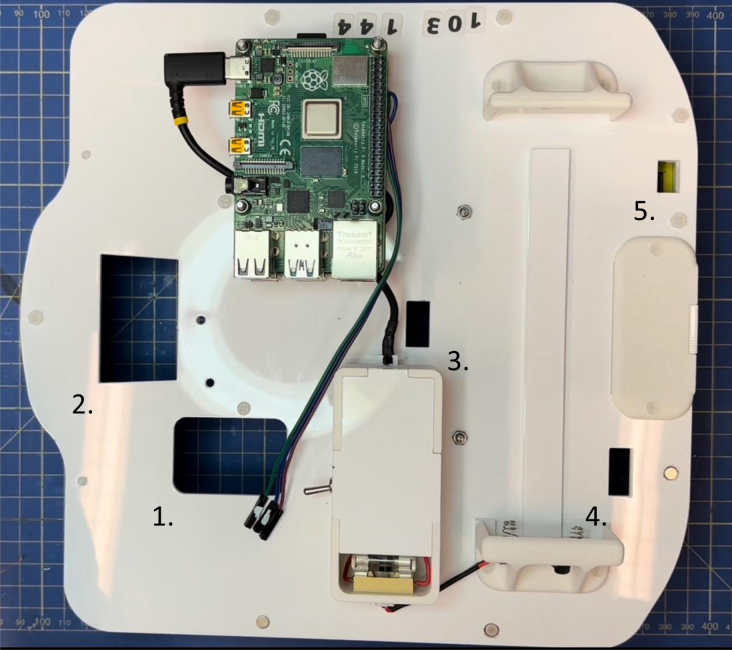
Cable apertures on the robot base plate. Each aperture (1, 2, 3, 4 and 5) is labeled according to its function and position to facilitate cable routing.

The following steps describe the setup of all internal connections required for power and data transmission between the various components of the robot. Each cable is routed early in the assembly process to ensure clean integration, mechanical safety, and accessibility. Cable apertures are carefully planned to minimize interference and are illustrated in Fig. 4.

**Fig. 5:**
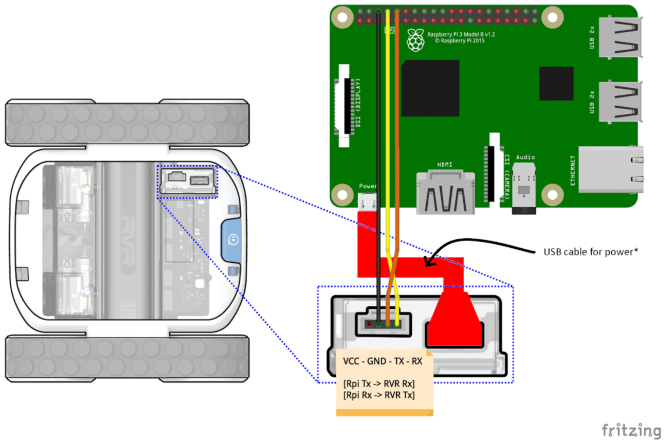
Wiring diagram [31] showing the power and serial data connections between the Raspberry Pi and the Sphero RVR+. The USB cable provides power, while jumper wires connect the GPIO pins for UART communication (TX/RX). Ensure correct orientation: Raspberry Pi TX to RVR RX, and Raspberry Pi RX to RVR TX.

1. **Inter-board communication setup:** Connect the two Raspberry Pi boards using an Ethernet cable (M25) to enable direct communication between them during robot operation.
2. **OAK-D camera routing:** Connect the OAK-D W camera to the upper Raspberry Pi using its USB-A to USB-C cable. Route the cable through the dedicated opening in the base plate (aperture **1** as indicated in Fig. 4) to guide it underneath the platform and up to the camera mount (aperture **2** as indicated in Fig. 4). This ensures a clean and protected cable path.
3. **IR sensors routing:** Connect the USB-A to micro-USB cable to the lower Raspberry Pi, then route it through the same opening in the base plate used for the camera (P9) (aperture **1** as indicated in Fig. 4). Guide the cable underneath the platform and bring it back up through the designated opening near the rear section of the base (aperture **4** as indicated in Fig. 4). Finally, pass the cable through the dedicated slot in the base to reach and connect the sensor belt (P1) (aperture **5** as indicated in Fig. 4).
4. **Fisheye camera cable routing (P12):** Connect the flat ribbon cable (FFC) from the upper Raspberry Pi’s CSI port and route it through the same opening in the base plate used for the camera and LiDAR cables (aperture **1** as indicated in Fig. 4). Guide the cable underneath the platform and bring it back up through the dedicated opening near the rear section of the base (aperture **4** as indicated in Fig. 4). The cable will be connected to the fisheye Pi Camera once the sensor tower is installed.
5. **LiDAR and LEDs cable routing:** Connect the power and data cable to the lower Raspberry Pi, then route it through the same opening in the base plate used for the OAK-D camera cable (aperture **1** as indicated in Fig. 4). Guide the cable underneath the platform and bring it back up through the second dedicated opening near the sensor tower mount (aperture **3** as indicated in Fig. 4). The LEDs jumpers can be directly connected to the appropriate pins of the upper Raspberry Pi (see Fig. 4).
6. **UWB module routing:** Connect the USB-A to micro-USB cables from the lower Raspberry Pi to each UWB module. Route the cables through the same opening in the base plate used for the other components (aperture **1** as indicated in Fig. 4), then guide them underneath the platform and connect them to the UWB modules.
7. **Base plate**Ensure that all routed cables pass neatly between the Sphero mounting plate and the base plate. This positioning helps to hold the cables in place, preventing them from shifting or getting caught in the robot’s moving parts during operation.
8. **Power and data connection to Sphero RVR+:** Once the base plate is securely attached to the Sphero RVR+, connect the lower Raspberry Pi to the robot using a USB-A to USB-C cable for power delivery (aperture **1** as indicated in Fig. 4). Additionally, establish the data connection using jumper wires between the Raspberry Pi’s GPIO pins and the Sphero’s serial interface (aperture **1** as indicated in Fig. 4). A detailed illustration of the power and data connections, including the pin mappings on both the Raspberry Pi and the robot, is provided in Fig. 5 to assist with the wiring process. Finally, connect the upper Raspberry Pi to the fuse case using the dedicated modified power cable (see Section 5.2.2).

### 5.3 Mercator-lite assembly

The Mercator-Lite variant follows a more compact assembly process, requiring only the materials listed in Table 6. Only the camera mount (P6) needs to be 3D-printed. Components are mounted directly onto the RVR or RVR+ mounting plate:

1. **Camera mount:** Attach the OAK-D camera (P11) to the Lite version of the camera mount (P6) and secure it on the mounting plate .
2. **LiDAR installation and connection:** Mount the YDLIDAR (P10) on the mounting plate using spacers (M13) attached two by two.
3. **Install Raspberry Pi board:** Mount the single Raspberry Pi 4 (P9) using spacers (M12).
4. **Power and data connection to Sphero RVR+:** Establish the data connection using jumper wires between the Raspberry Pi’s GPIO pins and the Sphero’s serial interface. A detailed illustration of the power and data connections, including the pin mappings on both the Raspberry Pi and the robot, is provided in Fig. 5 to assist with the wiring process.

## 6 Operation instructions

To operate Mercator, users can either rely on the pre-configured SD card images provided in Table 3 (S8–S10), or follow a step-by-step installation procedure to configure the software stack from scratch. This section presents both options. Before proceeding, ensure that the platform has been assembled and wired as described in Section 5.

### 6.1 Initial configuration and setup

#### 6.1.1 Option 1: Use of pre-built images

The fastest way to begin using the Mercator platform is to flash the corresponding SD card image (see Table 3 S8–S10) to each Raspberry Pi and boot the system. These images contain all necessary drivers, ROS nodes, and pre-installed repositories. After flashing and booting, only network configuration may be required:

**Figure:**
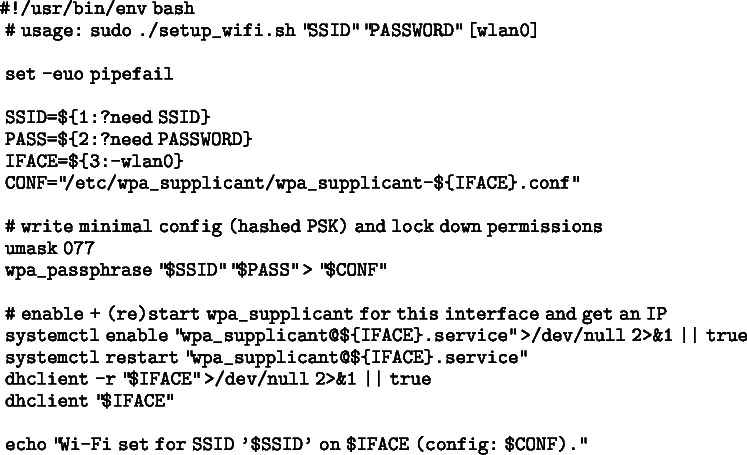

To streamline the setup of communication between the two Raspberry Pi boards, a Bash script was created to automatically configure the network interface and ROS environment variables. This script appends the necessary commands to the /.bashrc file — only if they are not already present — ensuring that the configuration is persistent across terminal sessions. Specifically, the script:

• Activates the Ethernet interface (eth0) and assigns it a static IP address (10.66.66.1 for the upper Raspberry and 10.66.66.2 for the lower Raspberry),
• Sets the ROS_MASTER_URI and ROS_IP environment variables required for ROS to function correctly in a multi-device setup.

This automation reduces the risk of manual errors and ensures consistent configuration, which is essential for reliable inter-device communication in ROS-based systems. The script for the upper Raspberry Pi can be found below:

**Figure:**
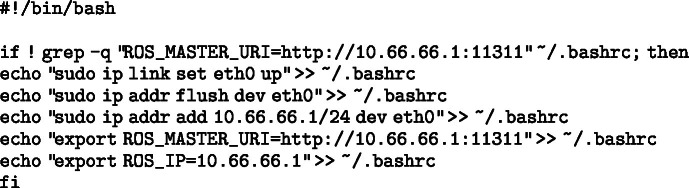

**Important:** No additional installation steps are required unless new components or updated software are introduced.

#### 6.1.2 Option 2: Build from scratch

If building from scratch, the following procedure installs all the necessary software components on a clean Ubuntu 20.04 system (tested on Raspberry Pi OS 64-bit).

##### 1. Flash ubuntu 20.04 to SD card.

Before installation, a fresh operating system image must be flashed onto each Raspberry Pi’s microSD card. We recommend using the official 64-bit Ubuntu Server 20.04 LTS for Raspberry Pi:

• Download from: https://ubuntu.com/download/raspberry-pi
• Flash the image using tools like:**Raspberry Pi Imager:** https://www.raspberrypi.com/software/

After flashing, insert the card into the Raspberry Pi, boot it, and proceed with initial setup (create a user, set up SSH/Wi-Fi, expand file system, etc.). Then update the system:

**Figure:**
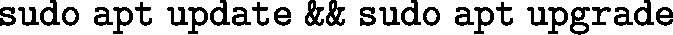

##### 2. Install ROS noetic.

Follow the official instructions or run:

**Figure:**
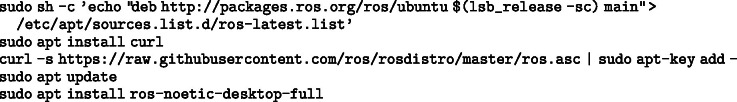

##### 3. Setup catkin workspace.

**Figure:**
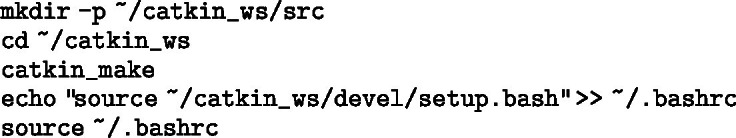

##### 4. Install the rvr_ros package.

**Figure:**
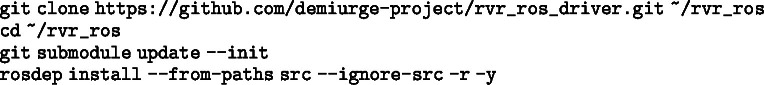

Then build:

**Figure:**
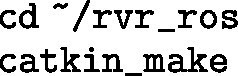

**Optional but recommended:** Add sourcing to .bashrc.

##### 5. LiDAR SDK.

**Figure:**
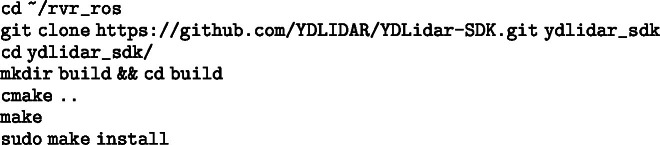

##### 6. Link Sphero SDK.

**Figure:**
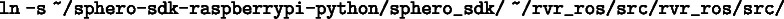

##### 7. Rename LiDAR port.

**Figure:**
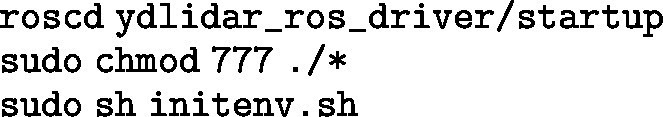

##### 8. Install ARGoS3 and the argos3-rvr plugin.

Install ARGoS3 beta48:

**Figure:**
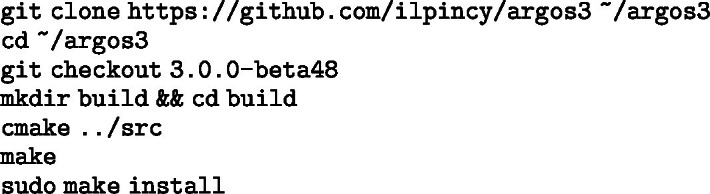

Then the plugin:

**Figure:**
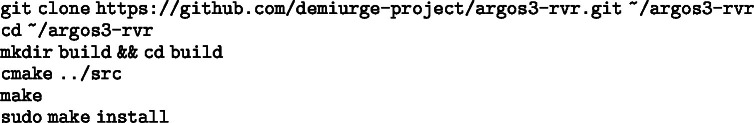

##### 9. Install DAO and AutoMoDe packages.

• RVR DAO:

**Figure:**
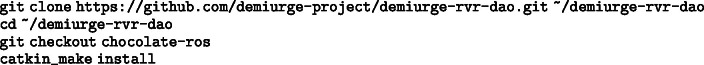
• Loop functions:

**Figure:**
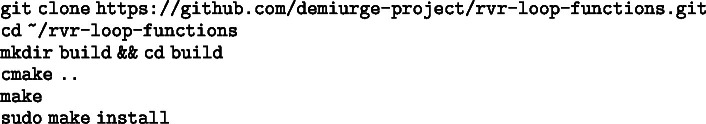
• AutoMoDe:

**Figure:**
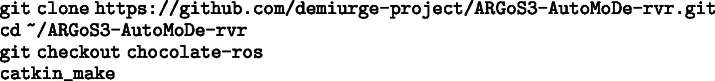
• NEAT:

**Figure:**
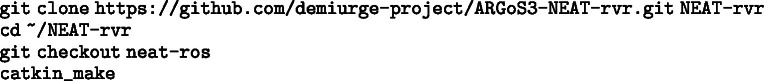

##### 10. Configure network and ethernet.

Use the scripts presented in Section 6.1.1 to configure network and ethernet connections.

### 6.2 Launching the system

Once setup is complete (via either option), the robot system can be launched in stages:

**Figure:**
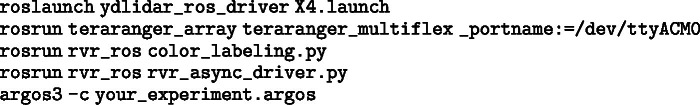

An example file is available in the experiments folder of the AutoMoDe repository. Be sure to update:

• Loop function path and label in loop_functions
• Controller path in automode_controller

**Stopping the robot:** The following command cleanly kills all ROS nodes and pythons scripts. It should be used before shutting down the robot, or for an emergency stop.

**Figure:**
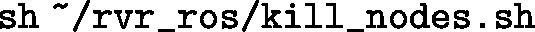

### 6.3 Mercator-lite setup

Mercator-Lite uses the same software stack, but only the primary Raspberry Pi is used. The same SD image (using S10) or manual setup instructions apply. Only the LiDAR and the OAK-D W camera nodes need to be launched, along with the minimal driver and control interface. Modules related to the secondary camera, LEDs, and proximity sensors can be ignored.

## 7 Validation and characterization

Mercator has been successfully developed, tested and validated both in simulation and in real-world scenarios. Experiments were performed to characterize its performance, robustness and suitability for research. The evaluation results are presented in this section.

### 7.1 Validation framework

The following tests can serve as standardized validation procedures for current and future versions of the Mercator platform. These tests aim to ensure reproducibility, reliability, and maintainability across deployments and hardware iterations.

#### 7.1.1 Battery life test

• **Test Purpose:** Measure operational time under predefined load conditions.
• **Procedure:** Fully charge the battery, then operate the robot continuously in a random walk while running all sensors until battery depletion.
• **Metrics:**
  – Time until RVR’s LEDs flash red (indicating that the battery is too low to ensure proper operation).
  – Time to full recharge.
• **Result:**
  – ∼2h before battery depletion (∼4h for Mercator-Lite).
  – Full recharge requires ∼1h per battery.

#### 7.1.2 Assembly robustness and repeatability

• **Test Purpose:** Evaluate the ease of assembly, risk of mechanical failure, and tolerances in repeated builds.
• **Procedure:**
  – Disassemble and reassemble the platform.
  – Log component fit issues, alignment errors, or failures.
  – Optionally, conduct shake/impact tests post-assembly.
• **Metrics:**
  – Assembly time.
• **Result:**
  – Assembly requires ∼1h per Mercator, and ∼15 min per Mercator-Lite.

### 7.2 Use case 1: Transferability experiments

Kegeleirs et al. [26] present an extensive empirical evaluation where control software and design methods initially developed for the e-puck platform were deployed on Mercator robots. These experiments validate:

i) the functional equivalence between the e-puck and the standard Mercator platform.
ii) the platform’s compatibility with automated design pipelines such as AutoMoDe and various neuroevolutionary strategies (NEAT, CMA-ES, xNES).
iii) the reliability of simulation-to-reality transfer for swarm behaviors with Mercator.

The results show that Mercator maintains meaningful collective behavior even when directly executing control software designed for another platform, and that it supports the direct application of full design methods without platform-specific tuning. This demonstrates not only transferability but also robustness to embodiment changes, further supporting the platform’s utility in reproducible swarm robotics research.

### 7.3 Use case 2: SLAM and inter-robot collaboration

In a separate project, we demonstrate the use of the Mercator-Lite variant in a heterogeneous multi-robot system. Several Mercator-Lite robots were equipped with SLAM capabilities and used its real-time mapping output to support the navigation of another robot in a shared environment.

• **Video demonstration:** https://www.youtube.com/watch?v=s-zTDPYx5do&t=2s
• **Capabilities demonstrated:**
  – Real-time SLAM and map sharing.
  – Inter-robot communication for assisted navigation.
  – Operation in dynamic and partially unknown environments.

This use case confirms that Mercator-Lite can serve as a mapping and coordination node within a heterogeneous robot team, opening the door for use in collaborative exploration, mixed-capability deployments, and swarm-to-multi-agent system bridges.

### 7.4 Use case 3: Education and research training

Mercator has been used in master’s thesis and students’ projects. Among others:

• Improving data sharing protocol in swarm SLAM [32].
• Developing communication protocols for Mercator [33].
• Enabling sensor fusion for other robots detection [34].

This use case demonstrates the suitability of Mercator for academic and educational applications. Its robust design ensures reliable performance even under varied experimental conditions, while its user-friendly interface makes it accessible to students with diverse technical backgrounds. The successful deployment of Mercators in multiple student-led initiatives highlights its practicality and ease of use, confirming its value as a dependable platform for research and hands-on learning.

### 7.5 Discussion

The structured validation framework proposed here supports reproducibility and quality assurance across different versions of the Mercator platform. Combined with the use cases presented above, these results highlight the platform’s ability to:

• Serve as a drop-in replacement for legacy platforms such as the e-puck, while offering modern sensing, compute, and software support.
• Participate in heterogeneous and collaborative robotics experiments, bridging swarm-specific research with broader multi-robot systems.
• Enable open benchmarking and repeatable experimentation in swarm and multi-agent robotics, thanks to its open-source design and simulation compatibility.

Beyond its direct role in swarm robotics, the Mercator platform can impact several other research areas:

• **Human–robot interaction (HRI):** With support for vision-based people detection and re-identification, Mercator can be used in studies of human–swarm collaboration, crowd assistance, and educational robotics.
• **Distributed AI and learning:** The modular dual-Raspberry Pi architecture makes it suitable for testing distributed reinforcement learning, on-board federated learning, and collective intelligence approaches.
• **SLAM and mapping research:** Its LiDAR–vision sensor combination enables robust experiments in collaborative SLAM and map sharing, especially in multi-robot exploration contexts.
• **Communication protocols:** The forthcoming UWB module bay and local communication capabilities make Mercator suitable for testing decentralized communication strategies, message-passing protocols, and robustness to packet loss.
• **Educational robotics:** The accessible, low-cost design allows students to engage with modern robotics concepts—ROS, SLAM, computer vision—while still being able to scale to swarm-level experiments.

While Mercator is not intended for commercial deployment, it is explicitly designed to provide swarm researchers with a platform that is compliant with modern mobile robotics capabilities, including navigation, mapping, and advanced perception. This design choice allows swarm robotics research to align with the expectations and standards of the broader mobile robotics community, from which it has historically diverged due to the reliance on ad hoc and highly simplified platforms such as Kilobot or e-puck. By adopting sensing modalities and software stacks commonly found in contemporary robotic systems—such as LiDAR, RGB-D cameras, and ROS-based architectures—Mercator facilitates the transferability of swarm algorithms and behaviors to other functionally equivalent platforms [26]. This transferability is particularly relevant for bridging swarm robotics research with larger-scale and commercial robotic systems, where comparable sensing and software infrastructures are already in place, thereby supporting a more direct pathway from laboratory swarm experiments to real-world robotic applications.

Future work will extend the Mercator family in several directions:

• **Hardware modules:** Development of new sensing and actuation modules (e.g., gas sensors for environmental monitoring, manipulator arms for collective transport, or additional depth sensors for 3D SLAM).
• **Communication systems:** Full integration of UWB and alternative radio systems to broaden support for large-scale experiments with realistic network constraints.
• **Design improvements:** Iterative redesign of the mechanical structure to reduce assembly time, improve durability, and further decrease cost—especially for the Lite variant.
• **Energy management:** Exploring higher-density or swappable battery systems to extend run-time without compromising payload capacity.
• **Software integration:** Expansion of ROS2 support, middleware for large-scale swarm orchestration, and standardized benchmarking pipelines for reproducibility.

By combining modularity, affordability, and advanced sensing, Mercator provides a foundation not only for addressing current challenges in swarm robotics but also for shaping future research in distributed autonomy, HRI, and AI-enabled collective systems. Its open design ensures that improvements by the community can feed back into the platform, strengthening its role as a long-term, shared resource for robotics research.

## CRediT authorship contribution statement

**Miquel Kegeleirs:** Writing – original draft, Visualization, Software, Methodology, Investigation, Conceptualization. **David Garzón Ramos:** Writing – original draft, Visualization, Software, Methodology, Investigation, Conceptualization. **Jeanne Szpirer:** Writing – original draft, Visualization, Validation, Software, Methodology, Investigation. **Cristobal Lara Vera:** Writing – review & editing, Validation, Software. **Guillermo Legarda Herranz:** Writing – review & editing, Software. **Ilyes Gharbi:** Writing – review & editing, Software. **Gianpiero Francesca:** Writing – review & editing, Resources, Conceptualization. **Mauro Birattari:** Writing – review & editing, Supervision, Resources, Methodology, Conceptualization.

## Ethics statements

N/A

## Declaration of competing interest

The authors declare that they have no known competing financial interests or personal relationships that could have appeared to influence the work reported in this paper.

## Footnotes

2 https://sphero.com/products/rvr.

3 The motors of earlier revisions are known to struggle under the full sensor payload of the standard configuration, but they support the reduced payload of Mercator-Lite.

4 https://index.ros.org/p/ros1_bridge/.

5 Available on Zenodo.
